# Comparing the types of haemochromatosis- from genetics to clinics

**DOI:** 10.1038/s41431-026-02084-z

**Published:** 2026-03-25

**Authors:** Pragnya Srinivasamurthy, Kosha J. Mehta

**Affiliations:** 1 GKT School of Medical Education, Faculty of Life Sciences and Medicine, King’s College London, London, UK; 2 Centre for Education, Faculty of Life Sciences and Medicine, King’s College London, London, UK

**Keywords:** Diseases, Genetics

## Abstract

Haemochromatosis is a genetic disorder of iron homeostasis. It can be caused by mutations in genes encoding the iron-regulatory hormone hepcidin (*HAMP*), and/or genes that regulate hepcidin expression (*HFE*, *HJV*, *TFR2*), or a gain-of-function mutation in the gene encoding hepcidin receptor ferroportin (*FPN1/SLC40A1*). HFE-related haemochromatosis is prevalent predominantly in individuals of northern European descent. These mutations result in dysregulated levels or activity of hepcidin, leading to high iron-saturation of transferrin followed by progressive liver iron accumulation in the absence of anaemia. To enable and enhance the understanding of haemochromatosis in both researchers and prospective medics, this review collates and discusses the genetic basis and consequent pathophysiology of the different types of haemochromatosis within a single, comparative review. The discussion is supported by figures and a summary table that compares the haemochromatosis types for prevalence, clinical manifestations, primary organs affected, iron-related biochemical parameters and mechanisms of iron loading. Also, gain-of-function ferroportin mutation is compared to ferroportin disease, which is a loss-of-function ferroportin mutation, and shows a tendency to anaemia. Essentially, HFE-related haemochromatosis (common type) and TFR2-related haemochromatosis (rare type) show late-onset, milder and gradual iron loading, and often involve liver and joint damage. In contrast, HJV- and *HAMP*-related haemochromatosis (rare types) show severe and rapid iron loading in the first three decades of life, with notable cardiac and endocrine complications. Hepcidin levels are more markedly decreased in HJV-related haemochromatosis compared to HFE and TFR2 types. There are minimal to absent levels of hepcidin in *HAMP*-related haemochromatosis.

## Introduction

Iron has an indispensable role in many cellular processes. It is an essential part of several biological molecules, such as haem and iron-sulphur (Fe/S) clusters, which facilitate processes like respiration, cofactor biosynthesis, and nucleic acid replication and repair [1, 2], with the majority of iron present in the haemoglobin of erythrocytes [3]. There are three main ways in which iron can enter the circulation: i) via absorption of dietary iron by the gut enterocytes, ii) via the release of iron from the iron-recycling macrophages, and iii) via release of iron from the iron-storing liver hepatocytes [4].

### Significance of regulating body iron levels and the role of hepcidin-ferroportin axis in maintaining iron homeostasis

There are no physiological pathways for excreting excess iron. When in excess, iron accelerates the Fenton reaction, and thereby accelerates free radical production [5]. The Fenton reaction, involving Fe(II) and hydrogen peroxide, results in the catalytic formation of highly reactive oxygen species, such as hydrogen peroxide and hydroxyl radicals [6]. This causes oxidative stress and damages tissues at a molecular and cellular level through various mechanisms like apoptosis, necroptosis, and ferroptosis [5, 7]. Therefore, maintaining iron homeostasis in the body is essential, and this occurs at the level of iron absorption and release of iron from iron stores.

The iron-hormone hepcidin, primarily secreted by the liver, mediates systemic iron homeostasis by binding to ferroportin. Ferroportin is the only identified cellular iron exporter in mammals, which is highly expressed on the basolateral membranes of enterocytes, hepatic macrophages and hepatocytes [3, 8, 9]. When iron levels are elevated, hepatic hepcidin expression increases, and it is secreted into the circulation. Hepcidin binds to ferroportin on the surfaces of various cell types and induces the internalisation, ubiquitination, and degradation of both ferroportin and hepcidin within the cells. This reduces ferroportin expression on cell surfaces, thereby inhibiting and/or limiting cellular iron export and preventing further iron influx into the circulation [4, 10]. When circulating iron levels are low or reduced, hepatic hepcidin expression decreases. Less hepcidin is synthesised and secreted into the circulation, and thus, less hepcidin is available to bind to ferroportin. This prevents the excessive degradation of ferroportin on cell surfaces, allowing iron export into the circulation. As a result, systemic iron levels are elevated, and thus, iron homeostasis is maintained systemically [11].

### Regulation of the iron-hormone hepcidin - the iron context

The control of hepcidin expression is fundamental in iron homeostasis [4]. The cellular mechanisms in the hepatocytes that induce hepcidin expression are still being understood.

In the context of induction by iron, hepcidin expression in liver hepatocytes is induced by two main triggers: elevated systemic iron levels and elevated tissue iron levels. These triggers involve two pathways that are mediated by several iron-related genes/proteins and have different starting points on the hepatocyte cell surface, but a common intracellular pathway that induces hepcidin transcription within hepatocytes. Participating proteins at the starting points include HFE, transferrin receptor-2 (TFR2), hemojuvelin (HJV), and bone morphogenetic receptors (BMPRs) found on hepatocyte cell surfaces.

In the pathway involving a hepcidin response to high systemic iron levels, transferrin, the iron-carrying protein in the circulation, plays a role. Upon elevation of systemic iron levels, iron-saturated transferrin (holotransferrin) binds to its receptor, transferrin-receptor-1 (TFR1). This dissociates HFE from its previously bound state with TFR1 on the hepatocyte cell surface. This dissociated HFE can form a complex with TFR2 (HFE-TFR2 complex) and trigger a cascade of intracellular events leading to hepcidin induction in hepatocytes. Holotransferrin can also bind to TFR2, which stabilises TFR2 and thereby facilitates its iron-sensing and hepcidin-inducing function. On the other hand, in the pathway involving hepcidin response to high tissue iron levels, there is increased secretion of bone morphogenetic protein 6 (BMP6) by hepatic sinusoidal epithelial cells. BMP6 interacts with BMP receptors, which are in complex with HJV (BMPRs-HJV) on the hepatocyte cell surface to eventually induce hepcidin expression. As indicated above, TFR2 and HFE can regulate hepcidin expression independently of HJV and BMP6. Formation of several multiprotein complexes on the hepatocyte cell-surface is thought to participate in hepcidin induction, for example, the HFE-TFR2-HJV complex, the HFE-BMPRs interaction and/or the HJV-BMPRs complex. The HFE-TFR2-BMPR complex can induce hepcidin expression (in the absence of BMP6, likely in the presence of BMP2 or 4), independent of hepcidin induction by the HJV-BMPR complex [4, 12–17].

These iron-induced induction pathways on the cell-surface seem to initiate the same/similar intracellular signalling pathway, known as the BMP-SMAD pathway in hepatocytes, through the phosphorylation of SMAD proteins (Smad-1, Smad-5, and Smad-8) in the cytoplasm [12, 18]. These phosphorylated SMAD protein complexes are then translocated into the nucleus and bind to BMP-responsive elements of the *HAMP* gene promoter (*HAMP* encodes the hepcidin peptide), thereby resulting in the upregulation of hepcidin expression and the consequent increase in hepcidin secretion in the circulation [12]. Thus, proteins such as HFE, TFR2, HJV, and BMP6 play a fundamental role in the upregulation of hepcidin expression. Studies indicate that HJV and BMP6 are more robust inducers of hepcidin expression in comparison to HFE and TFR2, which are relatively mild inducers [18]. This is particularly relevant in the context of distinct mutations implicated in the different types of haemochromatosis and their associated pathophysiological consequences.

### Rationale and aims

Mutations in the genes of these above proteins result in decreased *HAMP* (hepcidin) transcription and a consequent iron overload. However, there is variation in how the different mutations manifest, thus giving rise to different types of haemochromatosis [19]. While literature exists on the different types of haemochromatosis, it is often difficult to find literature that systematically compares haemochromatosis types in all contexts, namely, genetics, epidemiology, biochemistry, mechanism of iron accumulation, clinical onset and manifestations, and presents these within a single, comparative review.

This review aims to fill this gap, collate and discuss the aforementioned parameters, i.e., the genetic basis and consequent pathophysiology of different types of haemochromatosis in one article to enable and enhance the understanding of this genetic iron disorder in both researchers and prospective medics.

## The genetics and definition of haemochromatosis

### The genetics

Mutations in the gene encoding hepcidin or the genes that regulate hepcidin expression cause insufficient hepcidin production, and lead to the iron-loading condition called haemochromatosis. Also, mutations in the gene that affect hepcidin activity/action can lead to iron-loading haemochromatosis. Collectively, at least five genes are involved [20]. The mutation generally classifies the haemochromatosis type: HFE-related haemochromatosis and non-HFE-related haemochromatosis. HFE-related haemochromatosis is a more common type that is caused by a mutation in the *HFE* gene, affecting the HFE protein. The non-HFE-related haemochromatosis encompasses other mutations, i.e., mutations in genes encoding HJV (*HFE2*), hepcidin (*HAMP*), transferrin receptor 2 (*TFR2*), and ferroportin *(FPN1/SLC40A1)* [4, 21].

### Haemochromatosis definition and clinical manifestations

As per the European Association for the Study of the Liver (EASL), haemochromatosis is a genetic disorder of iron homeostasis, characterised by elevated intestinal iron absorption leading to elevated iron-saturation of the iron-carrier protein in circulation, transferrin (TSAT), and iron loading mainly in the liver (not in the spleen), in the absence of anaemia or reticulocytosis. Knowledge of splenic iron is helpful in the evaluation of extrahepatic manifestations of haemochromatosis and in distinguishing it from differential diagnoses where there is iron retention in splenic macrophages; this will be addressed in later sections of this paper. Haemochromatosis shows elevated serum ferritin/serum iron levels, prominent involvement of periportal hepatocytes, but without much iron loading in Kupffer cells, along with signs and/or symptoms associated with excess body iron [20–22].

The clinical manifestations of haemochromatosis vary according to the type of disease and are dependent on the degree of iron overload and the severity of organ damage. In some types, particularly HFE-related haemochromatosis, the condition is usually asymptomatic in its earlier stages [21]. When present, manifestations across different haemochromatosis types may include liver disease, joint disease, cardiomyopathies, diabetes, hypopituitarism, hypogonadotropic hypogonadism, chronic fatigue and mental health changes [21, 23]. Marked hyperpigmentation of the skin can be a common and early clinical manifestation of haemochromatosis. It is evident in sun-exposed areas of skin and may be accompanied by other cutaneous manifestations such as ichthyosiform changes (having dry, thick, scaly or rough skin) and skin atrophy (skin thinning) on the anterior aspects of the legs [22]. Hepatic involvement is common, with liver function abnormalities present in 75% of affected individuals. Clinical manifestations of liver disease may include jaundice, abdominal pain, hepatomegaly, and splenomegaly [22]. Progressive hepatic iron overload can cause liver fibrosis and cirrhosis, which may be further complicated by portal hypertension and ascites, primary liver cancer such as hepatocellular carcinoma, and end-stage liver disease, possibly resulting in liver failure or multi-organ dysfunction [24]. The arthropathy in haemochromatosis is similar to osteoarthritis; however, it typically has an earlier onset and affects the 2nd and 3rd metacarpophalangeal joints and ankles, and x-rays show degenerative changes with subchondral cysts, narrowing of joint space, and chondrocalcinosis [21]. Note that each haemochromatosis type varies in clinical presentation and does not necessarily display all of these clinical features described above.

The pathophysiological acquisition and accumulation of iron, which is typically genetically inherited, is also referred to as primary iron overload [4, 9, 21]. This is different from secondary iron overload, which occurs in haemoglobinopathies, diseases, such as alcohol-associated liver disease, acute viral hepatitis, metabolic-associated fatty liver disease and inflammatory diseases, such as rheumatoid arthritis. Secondary iron overload can also result from ingested iron-loaded foods and iatrogenic causes such as iron injections, infusions, and blood transfusions [24, 25].

### Digenic inheritance

Haemochromatosis may show a digenic inheritance. This means that in some individuals, there can be a mutation in not just one gene, but two iron-related genes. For example, individuals may have the C282Y variant of *HFE* in addition to heterozygous mutations in *HFE2*, *HAMP* or *TFR2* [20]. This could explain the phenotypic heterogeneity seen in haemochromatosis and explain why some patients with suspected haemochromatosis cannot be ascribed to any of its classifications [20, 26]. As such, haemochromatosis should no longer be considered as a monogenic disease but rather an oligogenic disorder [26].

### Another gene variant in iron overload- *BMP6*

Some individuals with iron overload do not possess variants in any of the aforementioned genes. The *BMP6* gene encodes for one of the main proteins involved in the upregulation of hepcidin expression in response to tissue iron overload. Recent studies have reported moderate late-onset iron overload in individuals with variants in the *BMP6* gene [20]. However, this is not yet classified as haemochromatosis and the role of such variants is controversial. Nonetheless, such variants broaden the spectrum of genetic mutations that can be included within the overarching term haemochromatosis [20].

## General overview of the prevalence and epidemiology of haemochromatosis

HFE-related haemochromatosis is the most common genetic disease in northern European populations and is almost exclusively found in Caucasian populations, with a prevalence of 0.3–0.5%. Generally, it is less prevalent in Asian, African and Hispanic populations. Non-HFE-related haemochromatosis can affect both Caucasian and non-Caucasian populations [4, 27, 28].

In haemochromatosis, males are affected two to three times more often than females. Usually, the disease biochemically and symptomatically manifests more often, earlier in life, and to a greater degree for males in comparison to females. This difference is possibly due to the blood loss and consequent iron excretion associated with menstruation and pregnancy in females, the antioxidant effect of oestrogen, and sex-specific HFE and non-HFE genetic modifiers. Genetic studies have shown that males homozygous for the D6S105 allele 8 on chromosome 6 had a predisposition towards greater iron loading, however, this effect was not observed in females [4, 22, 29]. Notably, serum ferritin levels increase after menopause [22].

## HFE-related haemochromatosis

The HFE protein is an atypical MHC class I protein expressed primarily in crypt cells of the duodenum, Kupffer cells of the liver, and macrophages. In addition to regulating transferrin-mediated cellular iron uptake, it is involved in the iron-sensing mechanisms and hepcidin upregulation, as discussed previously [26, 30].

HFE-related haemochromatosis involves a mutation in the *HFE* gene on chromosome 6, the most common mutations being C282Y and H63D, and is inherited in an autosomal recessive pattern [4, 20]. These mutations account for the majority of haemochromatosis cases and the condition is found almost exclusively in Caucasian individuals, especially in those of northern European descent [4, 22]. The majority of these patients are homozygous for the C282Y mutation, and a high proportion of the remaining patients are compound heterozygotes for C282Y and H63D [26]. HFE-related haemochromatosis is more severe in males and usually presents in adulthood with liver damage and arthropathy [20]. Hepatic fibrosis and cirrhosis are common, and hepatocellular carcinoma is the most frequent cause of death in these patients [31].

### C282Y mutation of *HFE*

The C282Y mutation of *HFE* results in the disruption of a disulphide bond within HFE protein, which changes the conformation of this protein. The mutant HFE protein collects in the Golgi of the cell and is rapidly degraded, thus, it fails to reach the cell surface efficiently [26]. This results in loss of functional HFE protein on the cell surface, diminishing the ability of the HFE-TFR2 complex in hepatocytes to sense plasma iron, and hindering the subsequent BMP-SMAD pathway of hepcidin induction [12, 19, 32]. A consequent decrease in circulating hepcidin levels results in increased ferroportin-mediated iron export, causing progressive systemic and tissue iron loading [19, 32].

While C282Y homozygosity predisposes an individual to an elevated TSAT which is suggestive of iron overload, it is not sufficient on its own to result in the signs and symptoms of haemochromatosis [26]. Although many individuals with this genotype show the biochemical evidence of iron overload, only some develop symptoms, and even fewer progress to serious hepatic complications such as cirrhosis or hepatocellular carcinoma [33]. This highlights a possible role of additional factors, such as biological sex, alcohol consumption, and genetic or epigenetic factors that predispose to iron overload in conjunction with C282Y homozygosity [34].

### H63D mutation of *HFE*

The prevalence of the H63D mutation in the general population is high, and is even more common than the C282Y mutation, which indicates that it may be a benign polymorphic variant [21, 32]. Unlike C282Y, the H63D mutation appears to have arisen in different ethnic populations [32]. The H63D mutation of *HFE* is milder in iron loading compared to the C282Y mutation; the former shows only slightly elevated serum ferritin and TSAT and does not show clinically significant iron overload [26, 32]. This may be because, unlike the C282Y mutation, the H63D HFE protein variant is properly processed and expressed on the cell surface, and therefore may exhibit some iron-sensing functionality. However, the variant does lead to reduced iron-sensing, thereby reducing hepcidin induction [26, 35].

Compound heterozygosity for the H63D mutation with C282Y (C282Y/H63D) is found frequently in haemochromatosis patients, although it is insufficient for showing a severe iron overload phenotype, and thus it is less frequently associated with significant iron overload or liver disease. However, when affected individuals with this genotype have additional genetic or environmental risk factors for the development of liver disease, they can present with phenotypic haemochromatosis [21, 32, 33].

### Phenotypes of HFE-related haemochromatosis

HFE-related haemochromatosis is composed of three phenotypes as follows.

i) Clinical HFE-related haemochromatosis refers to patients with end-organ damage secondary to iron overload. Here, there is iron overload due to excess parenchymal free iron. Clinical manifestations commonly include arthropathy and signs of liver disease [21, 24].
ii) Biochemical HFE-related haemochromatosis refers to patients with elevated TSAT and serum ferritin in the absence of clinical signs and symptoms of iron overload [24].
iii) Non-penetrant HFE-related haemochromatosis refers to individuals with the C282Y mutation but without clinical signs and symptoms or biochemical indicators of iron overload. These patients may show elevated TSAT in the absence of elevated serum ferritin levels [24].

## Non-HFE-related haemochromatosis

Non-HFE-related haemochromatosis refers to iron loading caused by mutations in genes apart from *HFE*, namely, *HAMP*, *HFE2*, *TFR2* and *SLC40A1* [4].

### HJV-related haemochromatosis

HJV is a membrane protein mainly expressed on hepatocytes, where it acts as a BMP co-receptor and promotes hepcidin expression via the BMP-SMAD pathway [4, 18]. HJV-related haemochromatosis results from a mutation in the *HFE2* gene on chromosome 1, which encodes HJV protein HJV. It is inherited in an autosomal recessive pattern [18, 20]. Mutations in *HFE2* impair BMP receptor signalling, which disrupts the BMP-SMAD pathway for hepcidin induction. This leads to a significant reduction in circulating hepcidin levels, causing increased iron export into the systemic circulation [19].

### *HAMP*-related haemochromatosis

Hepcidin, the iron-regulatory hormone, is encoded by the *HAMP* gene on chromosome 19 [20]. Like the HFE, HJV and TFR2 types of haemochromatosis, *HAMP*-related haemochromatosis is inherited in an autosomal recessive pattern. It is rare [20]. While HJV-related haemochromatosis results from dysregulation of the signalling pathways necessary for hepcidin synthesis, *HAMP* mutations directly impair hepcidin production, leading to its nearly complete or complete absence in this type of haemochromatosis [19, 36]. This absence allows for unregulated, constant ferroportin expression and activity on cell surfaces, causing excessive iron export into the systemic circulation [4, 36, 37]. Note that missense mutations in *HAMP* could also result in iron overload due to non-functionality of hepcidin, rather than decreased hepcidin synthesis; the latter is the case with all other types of haemochromatosis [4]. Furthermore, when mutations of *HAMP* are associated with other haemochromatosis variants (digenic inheritance), these mutations worsen the iron overload [19].

### Similarities in the aforementioned HJV- and *HAMP*-related haemochromatosis

HJV- and *HAMP*-related haemochromatosis present with a similar phenotype [20]. For example, unlike HFE-related haemochromatosis, which has a more adult onset, HJV *and HAMP*-related haemochromatosis typically present earlier between the 1st and 3rd decade of life [20, 22, 38]. It should be noted that although these types tend to present earlier in life, some patients are diagnosed in adulthood [20]. Variants in the *HFE2* and *HAMP* genes cause a more severe iron absorption compared to that caused by *HFE* mutations [20]. This highlights the critical role of these genes in hepcidin induction and accounts for the early onset and severity of HJV and *HAMP* haemochromatosis types [19, 20].

In these haemochromatosis types, individuals may present with non-specific symptoms, such as fatigue, arthralgia (joint pain), and loss of appetite, and this is often mistakenly attributed to iron deficiency anaemia [31]. In adolescence and adulthood, endocrine involvement is noted, of which hypogonadotropic hypogonadism is a frequent presentation with low levels of the gonadotropins: luteinising hormone, follicle-stimulating hormone, and testosterone [31]. Hypogonadotropic hypogonadism in haemochromatosis may clinically manifest with erectile dysfunction, loss of muscle mass, and osteoporosis in men, and diminished libido, amenorrhoea (absence of menstruation), and infertility in women [24, 31]. Aside from this, fasting hyperglycaemia, glucose intolerance, or frank diabetes may be present [31].

Both HJV and *HAMP*-related haemochromatosis are characterised by cardiac involvement; myocardial iron accumulation causes the development of restrictive cardiomyopathies with early diastolic dysfunction, which may progress to dilated cardiomyopathy with impaired systolic function [21]. Deposition of iron may also involve the conduction system, especially the atrioventricular node [21]. This may present with arrythmias including extreme bradycardia, tachycardia, and supraventricular arrhythmias such as atrial fibrillation, as well as dyspnoea on exertion and heart failure [21, 31]. If left unmanaged, cardiac disease, particularly heart failure, becomes the leading cause of death in patients with these types of haemochromatosis [31].

### TFR2-related haemochromatosis

TFR2 is part of the iron-sensing complex on hepatocytes and is an inducer of hepcidin expression [12]. TFR2-related haemochromatosis involves mutations in the *TFR2* gene on chromosome 7, which encodes TFR2 protein. It is a very rare autosomal recessive type of haemochromatosis [20]. Mutations in the *TFR2* gene disrupt the interaction between holotransferrin and TFR2, as well as the interaction between TFR2 and HFE. This, in turn, impairs the hepcidin-inducing BMP-SMAD pathway, leading to reduced *HAMP* transcription and consequently low circulating hepcidin levels [12, 19, 36]. Downstream, this results in greater iron export into the systemic circulation, thereby leading to iron overload [4, 39].

### Comparison of TFR2-related haemochromatosis with other haemochromatosis types

TFR2-related haemochromatosis clinically resembles HFE-related haemochromatosis. However, it is regarded as an “intermediate” form between HFE*-*, HJV*-*, and *HAMP*-related haemochromatosis, in the context of the age of presentation [31]. For example, the age of clinical manifestations in TFR2-related haemochromatosis (young adulthood, between 3rd and 4th decade of life) is between the ages of the clinical manifestations observed in HFE-related (around 5th to 6th decade of life) and *HAMP/*HJV-related (1st to 3rd decade of life) haemochromatosis types [20, 22, 38]. Notably, there are several reports of children with this type of haemochromatosis, which suggests that other factors could contribute to the earlier onset and more severe disease [19, 20]. Cardiac and endocrine organ involvement is seen less frequently in TFR2-related haemochromatosis, compared to HJV- and *HAMP*-related haemochromatosis [19].

**Fig. 1:**
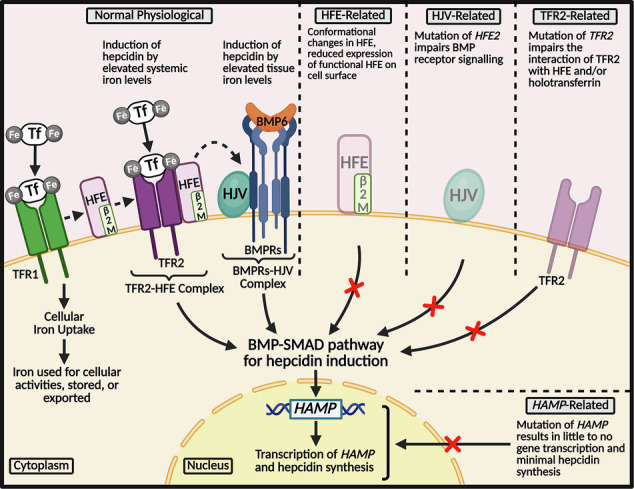
Overview of the mechanisms underlying the types of haemochromatosis. This figure illustrates the molecular mechanisms underpinning the different types of haemochromatosis. Under normal physiological conditions, the proteins HFE, TFR1, TFR2, HJV and BMP receptors expressed on the hepatocyte surface stimulate and regulate hepcidin expression via the BMP-SMAD signalling pathway, and thereby help in maintaining systemic iron homeostasis. In HFE-related haemochromatosis, mutations in *HFE* disrupt the formation of the iron-sensing complexes, including the HFE-TFR2 complex. In HJV-related haemochromatosis, mutations in *HFE2* impair BMP receptor signalling, which eventually hampers hepcidin induction. In TFR2-related haemochromatosis, mutations impair the interaction of TFR2 with holotransferrin and/or HFE. These disruptions to iron-sensing complexes lead to suppression of the BMP-SMAD pathway and decreased *HAMP* transcription and hepcidin synthesis. *HAMP*-related haemochromatosis involves mutations directly in the *HAMP* gene, leading to little or no hepcidin synthesis. Subsequently, decreased hepcidin levels lead to unregulated and constant ferroportin expression and activity, resulting in increased/excessive iron export into the circulation, ultimately causing iron overload. The dotted arrow represents the putative interactions between all the iron-sensing proteins/complexes [4, 12]. Abbreviations- β2M Beta-2 microglobulin, BMPRs bone morphogenetic protein receptors, BMP6 bone morphogenetic protein 6, Fe iron, HJV hemojuvelin, Tf transferrin, TFR1 transferrin receptor 1, TFR2 transferrin receptor 2. Created in BioRender. Srinivasamurthy, P. (2025) https://BioRender.com/dh4d4u7 for Fig. 1.

The Fig. 1 summarises the cellular mechanisms underlying the different types of haemochromatosis.

### Gain-of-function and loss-of-function mutations in ferroportin (*FPN1/SLC40A1*)

Hereditary iron overload disorders mentioned so far were due to loss-of-function mutations of *HFE*, *HFE2*, *HAMP* and *TFR2*. These haemochromatosis types share a) a similar pathophysiological consequence, which is impaired hepcidin induction, b) similarities in biochemical findings, e.g., elevated TSAT and serum ferritin, c) liver iron loading style i.e., iron accumulation in parenchymal cells without much iron loading in the Kupffer cells until late-stage disease, and d) clinical manifestations [38]. However, iron loading can also occur due to a mutation in the gene encoding the hepcidin receptor ferroportin (*FPN1/SLC40A1*). Unlike the aforementioned haemochromatosis types, here, not hepcidin induction but ferroportin is affected [38, 40].

The gain-of-function mutation of ferroportin (*SLC40A1*) is considered a haemochromatosis type, whereas the loss-of-function mutation of ferroportin (referred to as Ferroportin Disease) is not. The pathophysiological consequences of both gain-of-function and loss-of-function mutations of ferroportin are compared in Table 1.

**Table 1:** Comparison of gain-of-function and loss-of-function mutations of ferroportin (*SLC40A*1).

	Gain-of-function mutation of ferroportin (*SLC40A1)*	Loss-of-function mutation of ferroportin (*SLC40A1)* (Ferroportin Disease)
**Affected gene/protein**	• *FPN1/SLC40A1 e*ncodes the cellular iron exporter ferroportin, the receptor for the iron-hormone hepcidin [38]. *•* Heterozygote mutations on the *FPN1/SLC40A1* gene on chromosome 2 [4, 55]. *•* Mutations of other genes may be present alongside. The other genes may be associated with iron homeostasis, antioxidant defence, organ fibrosis, or some pathological conditions (e.g., viral hepatitis or metabolic syndrome). This may affect the phenotype [38].	
**Pattern of inheritance**	Autosomal dominant [38], unlike the other haemochromatosis types.	
**Condition type**	Primary iron overload [38].	
**Classification**	*• SLC40A1*-related Haemochromatosis [20]. *•* Otherwise known as FPN1-associated haemochromatosis [38]. *•* Previously classified as Type 4B haemochromatosis [38].	*•* Ferroportin Disease [20]. *•* Previously classified as Type 4A haemochromatosis [20].
**Rationale for classification**	Phenotypically and biochemically similar to hepcidin-deficient haemochromatosis (HFE-, HJV-, *HAMP-*, and TFR2- types) [20].	Phenotypically and biochemically different to the hepcidin-deficient haemochromatosis types. Characterised by distinctive features that do not fit the definition of haemochromatosis or its typical clinical picture. For example, it shows: *•* Normal to low TSAT. *•* Iron retention in the spleen and hepatic macrophages (Kupffer cells). *•* Sometimes, poor tolerance to phlebotomy management and/or mild anaemia following phlebotomy [20, 38, 56].
**Mechanism underlying pathophysiology and pattern of iron accumulation**	Gain-of-function variants in the *SLC40A1* gene affect the hepcidin binding site on ferroportin. This: *•* Renders ferroportin partially or completely resistant to the action of hepcidin [38]. *•* Prevents the internalisation, ubiquitination, and degradation of ferroportin [38]. *•* Increases ferroportin activity in enterocytes and macrophages, causing unregulated and constant cellular iron export in the systemic circulation [38]. *•* Leads to systemic iron overload, with iron accumulation in parenchymal cells, similar to that seen in HFE-related haemochromatosis [38, 41].	Loss-of-function mutations in *SLC40A1* either reduce ferroportin expression on the cell surface or reduce the iron export capability of ferroportin [20, 41]. *•* Iron is retained within cells, leading to tissue iron overload, particularly in non-parenchymal cells such as reticuloendothelial macrophages of the liver (Kupffer cells), spleen, and bone [20, 38]. *•* In some cases, a mixed form of hepatocellular and sinusoidal (Kupffer cell) iron overload has been reported [38]. *•* Hepatocellular iron overload may be present in the late stages of disease, although to a much lesser extent in comparison to the other types of haemochromatosis [38].
**Ferroportin’s iron export function**	Full iron export capability of ferroportin is exhibited [57].	Reduced/lack of iron export capability of ferroportin (or reduced ferroportin expression) is seen [57].
**Ferroportin’s response to hepcidin**	Hepcidin resistance (insensitivity to hepcidin action).	No hepcidin resistance.
**Hepcidin levels**	*•* Elevated levels of hepcidin in gain-of-function mutations of ferroportin [58].	*•* Inappropriately high levels of hepcidin can be an indicator of ferroportin disease [21]. *•* Low to normal (predicted) [58].
**Serum ferritin and iron levels**	Elevated serum ferritin level [38, 41] High serum iron level [38].	Low levels of circulating iron lead to: *•* A tendency to develop anaemia. *•* A tendency to develop iron-restricted erythropoiesis, especially when bone marrow demands are increased (e.g., menarche, phlebotomy) [20, 38].
**Transferrin saturation**	Elevated TSAT [41].	Normal to low TSAT due to the decreased availability of iron in the circulation to bind to serum transferrin [38, 59].
**Epidemiology and prevalence**	*•* Distributed worldwide, affects Caucasians and non-Caucasians (unlike HFE-related haemochromatosis) [38]. *•* Highest prevalence among Africans [20].	
**Epidemiological feature**	Some gain-of-function variants of *SLC40A1* could be the commonest genetic cause of hepatic iron overload in non-Caucasian individuals with the typical presentation for haemochromatosis (e.g., elevated serum ferritin, elevated TSAT, iron accumulation within parenchymal cells causing damage and disease to target organs, etc.) [44].	Accounts for one of the commonest forms of hereditary iron overload disorder besides HFE-related haemochromatosis [38].
**Clinical onset**	4th-5th decade of life [38].	At any age [38].
**Clinical course**	*•* Mild to severe clinical course and phenotype [38]. *•* Similar to HFE-related haemochromatosis [41].	Mild clinical course and phenotype in comparison to haemochromatosis, due to the prevalent iron accumulation in macrophages, which are less prone to the toxic effects of iron in comparison to hepatocytes [20].

**Fig. 2:**
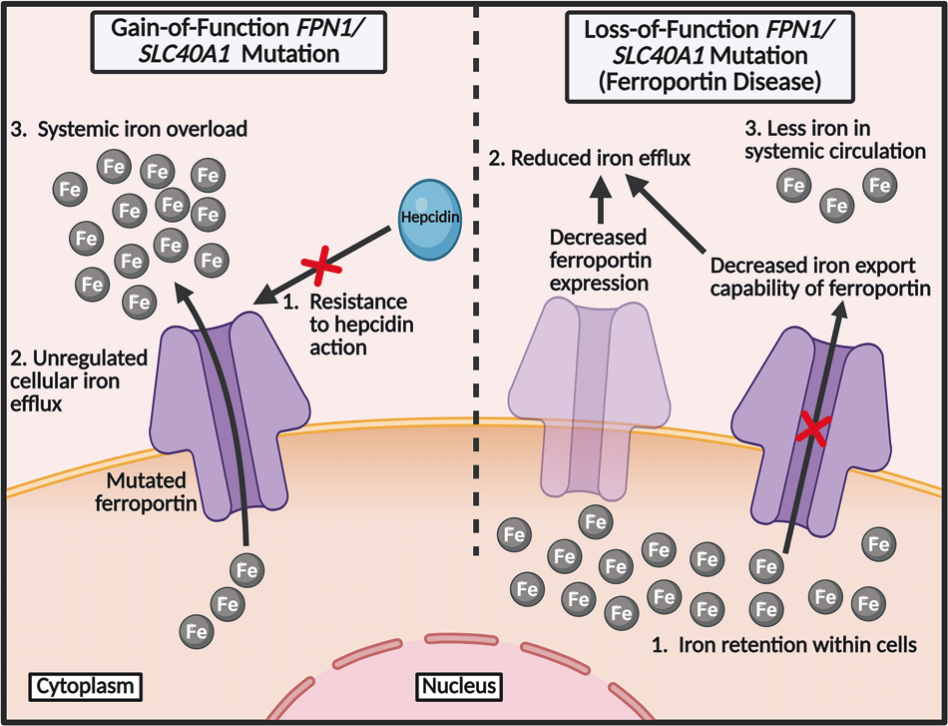
Overview of iron overload patterns due to mutations in *SLC40A1.* This figure illustrates the iron-related cellular mechanisms underpinning the two types of *SLC40A1* mutations. In gain-of-function *SLC40A1* mutations, ferroportin is resistant to hepcidin activity. This leads to unregulated iron export from enterocytes and macrophages and thus, systemic iron overload. In contrast, loss-of-function *SLC40A1* mutations (ferroportin disease) reduce ferroportin expression or function, i.e., the iron export capability. This impairs iron export from the mutated ferroportin, leading to intracellular iron retention (especially within macrophages) and thereby, low systemic iron levels. Created in BioRender. Srinivasamurthy, P. (2025) https://BioRender.com/5dgni1m for Fig. 2.

The Fig. 2 presents the cellular mechanisms underlying the cause of pathophysiology in the case of gain-of-function and loss-of-function mutations of ferroportin (*SLC40A1*).

## Common and different diagnostic investigations for the haemochromatosis types

### Generic biochemical parameters used to test all types of haemochromatosis

The initial approach to evaluating patients with suspected haemochromatosis is assessment of serum iron parameters, which mostly include TSAT and serum ferritin. These are elevated in all haemochromatosis types [21]. However, ferroportin disease differs from these aforementioned types as here, TSAT levels are normal to low, and anaemia may be present (Table 1) [41]. Hepcidin has a limited role in the diagnosis of haemochromatosis, because although its low levels are heavily implicated in its pathophysiology, no universal reference ranges have been established yet [21].

### Genetic testing for haemochromatosis

Patients with serum iron parameters suggestive of haemochromatosis should undergo genetic testing for the condition. The most common disease-associated genotype, particularly in Caucasians, is heterozygosity for the *p.C282Y* variant in *HFE*, so patients typically undergo genotyping for this initially [19, 21]. If patients are positive for this genotype, a diagnosis of HFE-related haemochromatosis is made [21].

In cases where HFE-related haemochromatosis has been excluded as a diagnosis, genotyping for the other types of haemochromatosis and Ferroportin Disease (including *HFE2, HAMP, TFR2 and SLC40A1*) is done, guided by clinical suspicion [20, 21]. The age at presentation may be a useful clinical indicator; for example, HJV- and *HAMP*-related haemochromatosis are more likely to be seen in younger patients, while TFR2-related haemochromatosis is more likely to be seen in older patients. However, very early or very late presentations are seen across all types of haemochromatosis [19]. A family history of dominant transmission is also a useful clinical indicator and thus prompts genetic testing for mutations in *SLC40A1* [19]. In these patients with elevated TSAT and elevated serum ferritin levels but with non-HFE genotypes, the diagnosis of haemochromatosis requires the presence of hepatic iron overload, either on MRI or liver biopsy [21].

All types of haemochromatosis discussed here are inherited disorders. Therefore, individuals with a first-degree family history of the disease should undergo genetic testing, followed by appropriate genetic counselling [21].

### Investigation of liver fibrosis in all haemochromatosis types

Hepatic iron overload and associated tissue damage is reflected by the degree of liver fibrosis, i.e., fibrosis indicates progressive liver disease. So, all individuals with haemochromatosis are assessed for the presence of liver fibrosis [21]. Liver fibrosis indices use routine blood test parameters to provide scores that help detect advanced fibrosis; for example, APRI (aspartate aminotransferase-to-platelet ratio index) and FIB-4 (age, platelet count, aspartate aminotransferase, and alanine aminotransferase) [21].

Due to the use of genetic testing and the increasing use of non-invasive MRI, liver biopsy is rarely necessary to establish a diagnosis of haemochromatosis. However, it remains valuable for  a) assessing liver fibrosis or cirrhosis, b) distinguishing parenchymal from mesenchymal iron overload, and c) determining the presence and severity of co-existing liver disease, such as alcohol-associated liver disease or metabolic-associated fatty liver disease [19, 33, 42]. However, liver biopsy is invasive and presents a risk of complications such as pain, bleeding, biliary peritonitis, and pneumothorax [43]. Alongside, non-invasive methods have gained popularity in recent times, which include the usage of MRI and transient elastography for assessing liver disease. Therefore, a biopsy is usually reserved for circumstances where other methods/approaches are unavailable, contraindicated (such as in the presence of coagulopathy, thrombocytopenia, and ascites), or in individuals with suspected advanced liver disease, as discussed above [19, 33, 42, 43].

### Differences between iron overload conditions in the context of hepatic iron loading and fibrosis progression

The mode of iron deposition and fibrosis progression differ between i) haemochromatosis, ii) Ferroportin Disease, and iii) secondary iron overload seen with other liver diseases like chronic viral hepatitis or alcohol-associated liver disease [21]. For example, in the early stages of haemochromatosis, iron deposition is mainly present in periportal hepatocytes, which later extends to a panacinar distribution and even to bile duct epithelium, Kupffer cells, and portal macrophages in advanced cases. One key consequence of this is progressive periportal fibrosis [33]. In contrast, in Ferroportin Disease, iron deposition is predominantly in reticuloendothelial cells or both reticuloendothelial cells and hepatocytes, without a periportal dominance. On the other hand, in secondary iron overload seen with other liver diseases, iron deposition is usually mild and generally occurs in both perisinusoidal lining cells (Kupffer cells) and hepatocytes in a panlobular distribution [42].

### Investigation of extrahepatic manifestations in haemochromatosis

In severe or advanced haemochromatosis, cardiac and endocrine manifestations may be present. For example, cardiomyopathy and endocrine failure (mainly pituitary hypogonadism) are prevalent extrahepatic manifestations in HJV*-* and *HAMP*-related haemochromatosis. Clinical investigation of these includes evaluation of signs and symptoms of cardiac disease, ECG, Holter-ECG and transthoracic echocardiography. Cardiologist consultation is recommended for these individuals, and management should be initiated as per standard cardiology practice. Endocrine investigations include measurements of sex hormones, assessment for diabetes, and, rarely, thyroid, adrenal, and parathyroid status [21].

Generally, MRI is helpful in the clinical evaluation of extrahepatic manifestations of haemochromatosis and guiding appropriate management. In HJV*-* and *HAMP*-related haemochromatosis, cardiac MRI is useful in assessing cardiac iron accumulation and its pathophysiological consequences [21]. Spleen iron quantification by MRI may be useful in the diagnostics, because splenic iron is normal to low in haemochromatosis [21].

In haemochromatosis, joint disease and osteoporosis are common. Hence, joint concerns should be investigated and monitored where appropriate. This can be evaluated with X-rays and DEXA scanning for reduced bone mineral density [21, 31].

### Differential diagnoses for haemochromatosis

The differential diagnoses for haemochromatosis include conditions with similar clinical or biochemical findings, such as elevated TSAT or elevated serum ferritin.

For example, an elevated TSAT can occur in conditions where iron homeostasis is disrupted due to reasons beyond haemochromatosis. Atransferrinemia (a rare autosomal recessive disorder with severe deficiency in serum transferrin) shows elevated TSAT, parenchymal and diffuse iron accumulation, and iron deficiency anaemia [44]. Atransferrinemia can be distinguished from other types of haemochromatosis by its early onset (typically between one and two years of age), the presence of severe microcytic anaemia that may require blood transfusion, undetectable serum transferrin, very low serum iron levels, and the development of severe iron-related complications, which can be fatal [44]. Other differential diagnoses for an elevated TSAT include alcohol-associated liver disease, liver cirrhosis, frequent blood transfusions, dyserythropoiesis and iron-loading anaemias [20, 21].

Acute phase response is a systemic reaction to local or systemic infection, inflammation, and malignancy. While ferritin is the iron storage protein and is elevated during iron-loaded conditions, it is also an acute phase reactant, and it is elevated during the aforementioned acute phase responses. During such inflammatory states, elevated serum ferritin levels (hyperferritinaemia) do not correctly reflect body iron stores. Therefore, these pathological states should be considered as differential diagnoses for hyperferritinaemia alongside haemochromatosis [45, 46]. Additionally, hyperferritinaemia is often present in conditions associated with fatty liver disease (e.g., excess alcohol consumption and metabolic syndrome), so these are also differential diagnoses [21]. Another differential diagnosis for hyperferritinaemia is Gaucher Disease, a rare lysosomal disorder associated with iron overload. It typically presents with cytopenia, abnormal coagulation, hepatosplenomegaly, and neuropathic manifestations. The clinical manifestations of severe Gaucher Disease (with severe anaemia or extreme splenomegaly) resemble haemochromatosis, but can be investigated and distinguished through genetic testing [47, 48].

Ferroportin Disease typically presents with hyperferritinaemia but a normal to low TSAT (Table 1) [38]. It can be challenging to distinguish this disease from other causes for hyperferritinaemia (e.g., other liver diseases, Gaucher Disease, infection, inflammation, malignancy, etc) or causes of low TSAT (e.g., aceruloplasminemia) [44]. Thus, those differential diagnoses should also be considered and investigated.

Aceruloplasminemia is a rare autosomal recessive neurodegenerative disorder of iron overload, caused by a homozygous mutation in the ceruloplasmin gene, and it represents an important differential diagnosis for haemochromatosis and Ferroportin Disease [49]. Ceruloplasmin has an important role in maintaining iron homeostasis by functioning as a plasma ferroxidase, oxidising ferrous iron to facilitate its transfer to plasma apotransferrin and promoting the release of iron from endothelial storage sites [50]. Mutation in the ceruloplasmin gene results in the absence of functional ceruloplasmin and its ferroxidase activity [49]. This leads to an accumulation of ferrous iron in the plasma, which eventually gets deposited in the liver and other organs, comparable to what is observed in haemochromatosis [50]. Similar to Ferroportin Disease, aceruloplasminemia has features of marked increase in iron in both hepatic and reticuloendothelial cells, hyperferritinaemia, a mild anaemia, and a normal to low TSAT [44, 50]. Ceruloplasmin also has an important role in protecting the central nervous system from iron-mediated free radical damage. As such, Aceruloplasminemia is unique in that it involves both systemic and neurological iron accumulation. Clinically, this condition presents with signs and symptoms associated with iron overload in parenchymal tissues such as the liver, pancreas, and heart, as well as neurological manifestations like ataxia, involuntary movements, Parkinsonism, and cognitive dysfunction [49].

Hepcidin deficiency is a hallmark of HFE-, HJV-, *HAMP*- and TFR2-related haemochromatosis. But hepcidin deficiency is also observed in other clinical states, such as in cases of alcohol abuse and end-stage liver disease. So, such other causes of hepatic deficiency should be considered when investigating for haemochromatosis [20].

It is important to note that patients can present with co-existing conditions; the diagnosis of one does not exclude the other.

## Haemochromatosis management

### First and second-line management

The first-line management for all types of haemochromatosis is phlebotomy. The aim here is blood removal to stimulate erythropoiesis, thereby mobilising the iron and utilising excess iron for haem synthesis [21].

Phlebotomy management typically comprises two phases: an initial/induction phase and a maintenance phase. The induction phase can take several months or more, depending on the severity of iron overload, and typically involves weekly or biweekly phlebotomies. During the induction phase, the patient’s haemoglobin and serum ferritin levels should be monitored closely, as well as ahead of each phlebotomy. If haemoglobin levels are <12 g/dl, the rate of phlebotomy should be reduced, and if they drop <11 g/dl, then phlebotomy should be paused. Serum ferritin levels should be checked after four phlebotomies until a < 200 µg/L level is reached, then these levels should be checked every one to two phlebotomy sessions; the eventual target serum ferritin level during the induction phase is 50 µg/L [21].

The maintenance phase aims to prevent iron re-accumulation and involves infrequent phlebotomy sessions, typically two to six times a year. Like the induction phase, haemoglobin levels should be checked ahead of each phlebotomy, transferrin saturation and serum ferritin should be checked every six months, with a target serum ferritin level of 50–100 µg/L [21]. Phlebotomy management is typically carried out in a secondary healthcare setting and is quite intensive, so there are concerns regarding its potential adverse impact on patients’ lives [21, 51].

Phlebotomy is the management for Ferroportin Disease as well. However, these patients may have a lower tolerance for phlebotomy compared to the other hereditary iron overload disorders. This is because Ferroportin Disease tends to show macrophage iron overload, which is highly resistant to iron withdrawal. As a result, patients may develop anaemia before adequate depletion of iron stores is achieved. So, phlebotomy should not aim at reaching the usual targets for iron depletion, as for the other types of haemochromatosis, but instead, should be more conservative. Essentially, serum ferritin and TSAT should be even more closely monitored during the management of Ferroportin Disease [38]. Phlebotomy is not effective or recommended in patients with hyperferritinaemia or iron overload due to metabolic-associated liver disease [21].

For patients where phlebotomy may be contraindicated, such as anaemia, cardiac disease, or venous access issues, or in refractory cases, iron-chelation therapy may be considered as an alternative second-line management [21]. In HJV- and *HAMP*-related haemochromatosis, cardiac iron accumulation resulting in severe clinical manifestations is prevalent, so iron-chelation therapy is usually given alongside phlebotomies during the maintenance stage of phlebotomy. Iron-chelation agents include oral drugs such as deferasirox and parenteral drugs such as deferoxamine, which may be prescribed and administered following careful clinical consideration. This is because while these are effective in removing excess iron, there is limited experience with their use in haemochromatosis, and many patients experience side effects [21, 51]. It should be noted that all iron chelation drugs are contraindicated in pregnancy, and dose adjustment is necessary in those with renal failure [21].

### Management of haemochromatosis-induced complications

For patients with haemochromatosis and established liver cirrhosis, regular endoscopic evaluation and ultrasounds are necessary to monitor for potential complications, such as varices, portal hypertension, and hepatocellular carcinoma [24, 31]. Management for potential cirrhosis complications includes prophylaxis with non-selective beta-blockers and salt restriction and diuretics for ascites, with paracentesis and portosystemic shunts if necessary [24]. Phlebotomy is effective, so liver transplantation is not usually indicated as a management option for liver disease in haemochromatosis. Liver transplantation remains a surgical management option for end-stage liver disease and is effective for patients with decompensated cirrhosis and hepatocellular carcinoma [31, 52]. This is because while phlebotomy can improve liver fibrosis, even in advanced cases, it cannot reverse liver cirrhosis once it is established [31]. However, for haemochromatosis, liver transplantation is associated with poorer outcomes compared to transplantation for other causes of liver disease. This is mostly due to infection or cardiac-related complications in the former cases [52]. These complications are likely related to inadequate removal of excess iron stores before transplantation [31, 42].

Extrahepatic clinical manifestations and complications associated with haemochromatosis are managed as they normally would be. For example, hypogonadism is managed with appropriate hormone replacement therapy, and glucose intolerance and diabetes are managed with diabetic agents or insulin. Although phlebotomy is the mainstay for haemochromatosis, it does not resolve joint concerns/osteoporosis in these patients. Also, phlebotomy management does not prevent the development of joint disease in haemochromatosis patients. So, joint disease should be monitored and managed appropriately. This includes usage of analgesics, non-steroidal anti-inflammatory drugs, and orthopaedic procedures like joint replacement [21, 31].

In some patients, the additional use of proton pump inhibitors (PPIs) may be helpful in their management, as it reduces non-haem iron absorption by increasing the gastric pH [21]. Some studies have shown a reduction in the need for phlebotomy in patients taking PPIs [53]. However, the effectiveness of PPIs has not been fully studied, and so, it should be considered as part of supportive management, not as part of first or second-line management for haemochromatosis [21].

As low/absent hepcidin activity has been implicated in the pathophysiology behind most haemochromatosis types, hepcidin analogues have been proposed as potential management. However, as currently formulated, these drugs are likely to be parenteral and more costly in comparison to phlebotomy [4].

Supportive management and patient counselling are important aspects of the management of haemochromatosis. For example, patients should be given lifestyle advice including dietary modifications, to reduce long-term iron accumulation and potential complications [21]. Alcohol consumption should be limited or completely avoided, as this has been associated with a high risk of liver fibrosis, cirrhosis and cancer in patients with haemochromatosis [21, 54]. Vitamin C increases the bioavailability of non-haem iron for enteric absorption and has also been associated with the acceleration of iron deposition and acute deterioration of cardiac function, likely due to iron mobilisation, increased iron availability and free radical generation. Thus, patients should be counselled to avoid vitamin C and iron supplementation [21].

## Summary

Table 2 summarises the epidemiological, genetic, mechanistic, biochemical and clinical features of different types of haemochromatosis.

**Table 2:** Core features of the different types of haemochromatosis.

	Normal	HFE-related haemo-chromatosis	HJV-related haemo-chromatosis	*HAMP*-related haemo-chromatosis	TFR2-related haemo-chromatosis	*SLC40A1*-related haemo-chromatosis (Gain-of-function variants of *SLC40A1*)	Ferroportin disease (Loss-of-function variants of *SLC40A1*)
**Genetics**	N/a	Autosomal recessive [20]				Autosomal dominant [20, 38]	
**Epidemiology**	N/a	Highest prevalence in northern Europe [20]	Highest prevalence in Southern Asia [20]	Several populations [20]	Most frequent among non-Finnish European populations [20]	Distributed worldwide, affects Caucasians and non-Caucasians [38]	
**Serum iron**	Female: 11–29 µmol/L Male: 14–31 µmol/L [60]	Elevated [21, 41]					Normal to low [38]
**TSAT**	20–40% [20]	Elevated; Female: >45%, Male: >50% [20, 21]					Normal to low [38, 59]
**Serum ferritin**	Female: 30–200 µg/L Male: 30–300 µg/L [20]	Elevated; Female: >200 µg/L, Post-menopausal female and male: >300 µg/L [21]					
**Serum hepcidin levels (in comparison to the normal range)**	Normal range [4]	Low [58]	Very low [58]	Very low or absent [36, 58]	Low [58]	Elevated [58]	Inappropriately high levels can be an indicator [21] Low to normal (predicted) [58]
**Iron loading style and severity**	N/a	Mild and gradual iron loading [44, 61]	Massive, rapid, and most severe iron loading [61, 62]		Gradual iron loading [61]. More severe iron overload in comparison to HFE type [19]	Phenotypically and biochemically similar to HFE and TFR2 types [19, 20]	Moderate to severe iron loading [34]
**Mechanism of iron accumulation**	N/a	Disruption of hepcidin induction results in increased ferroportin-mediated iron export into the systemic circulation, with iron accumulation in parenchymal cells [32]				Resistance of ferroportin to hepcidin action, resulting in unregulated/constant iron export into the systemic circulation, with iron accumulation in parenchymal cells [38, 41]	Decreased ferroportin expression or its reduced iron export capability results in decreased cellular iron export, leading to tissue iron overload with iron accumulation in non-parenchymal cells [20, 38, 41]
**Clinical onset age**	N/a	4th to 5th decade of life [38]; 5th to 6th decade of life [22]	1st to 3rd decade of life [22, 38]		3rd to 4th decade of life [38]	4th to 5th decade of life [38]	At any age [38]
**Clinical manifestations (hallmark) in addition to liver disease**	N/a	Arthropathy [20]	Hypogonadotropic hypogonadism, cardiomyopathy [31]		Arthropathy [20]	Arthropathy [38]. Iron deposition predominantly in hepatocytes [41].	Anaemia [38]. Iron deposition predominantly in macrophages and the spleen. Poor tolerance to phlebotomy [20, 41].
**Clinical course**	N/a	Mild to severe [38]	Severe [38]		Mild to severe [38]	Mild to severe [38, 41]	Mild [38]

Essentially, HFE and non-HFE-related haemochromatosis are caused by different genetic mutations, exhibiting variations in severity of disease, clinical presentation, investigation, and management. Below are a few important points.

1. HFE-related haemochromatosis is highly prevalent among individuals of northern European descent, while non-HFE-related haemochromatosis is more common worldwide.
2. HJV- and *HAMP-*related haemochromatosis have a severe clinical course due to rapid and severe iron accumulation, while the HFE type has a mild to severe clinical course with slow and progressive iron accumulation.
3. HFE-related haemochromatosis presents in adulthood around the 5th and 6th decades of life with liver damage and arthropathy, as do gain-of-function ferroportin mutations, whereas HJV and *HAMP* types present between the 1st and 3rd decades of life with prominent endocrine and cardiac involvement.
4. TFR2-related haemochromatosis presents similarly to the HFE type, but between the 3rd and 4th decades of life, and therefore, it is considered to be an intermediate form of haemochromatosis between all of the types.
5. All haemochromatosis types show elevated TSAT and elevated serum ferritin levels.
6. Distinct from haemochromatosis, Ferroportin Disease shows normal to low TSAT and anaemia.
7. Haemochromatosis management may subtly differ depending on the degree of disease severity and organ damage. For example, phlebotomy alone may be insufficient for managing iron overload in HJV- and *HAMP*-related haemochromatosis, necessitating adjunctive iron chelation therapy. Organ damage is managed accordingly; for example, extensive liver damage may necessitate transplantation. Thus, recognising the differences between the types is crucial for an accurate diagnosis and personalised management to effectively manage iron overload.

## Data Availability

Not applicable.
